# HIV and Menopause: A Systematic Review of the Effects of HIV Infection on Age at Menopause and the Effects of Menopause on Response to Antiretroviral Therapy

**DOI:** 10.1155/2013/340309

**Published:** 2013-12-19

**Authors:** Kentaro Imai, Madeline Y. Sutton, Rennatus Mdodo, Carlos del Rio

**Affiliations:** ^1^Rollins School of Public Health, Emory University, Atlanta, GA 30333, USA; ^2^Division of HIV/AIDS Prevention, Centers for Disease Control and Prevention, Atlanta, GA 30333, USA; ^3^Epidemic Intelligence Service, Centers for Disease Control and Prevention, Atlanta, GA 30333, USA; ^4^Hubert Department of Global Health, Emory University, Atlanta, GA 30333, USA

## Abstract

More than half of persons living with HIV infection in the United States (U.S.) will be ≥50 years of age by 2020, including postmenopausal women. We conducted a systematic literature review about the effects of (1) HIV infection on age at menopause and (2) menopause on antiretroviral therapy (ART) response, in order to inform optimal treatment strategies for menopausal women living with HIV infection. We used the Ovid Medline database from 1980 to 2012. We included studies that focused on HIV-infected persons, included postmenopausal women, and reported outcome data for either age at menopause or response to ART across menopause. We identified six original research articles for age at menopause and five for response to ART across menopause. Our review revealed that current data were conflicting and inconclusive; more rigorous studies are needed. Disentangling the effects of menopause requires well-designed studies with adequate numbers of HIV-infected and HIV-uninfected women, especially disproportionately affected women of color. Future studies should follow women from premenopause through menopause, use both surveys and laboratory measurements for menopause diagnoses, and control for confounders related to normal aging processes, in order to inform optimal clinical management for menopausal women living with HIV.

## 1. Introduction

Globally, persons with human immunodeficiency virus (HIV) are living longer, healthier lives, due largely to the widespread use of effective highly active antiretroviral therapy (HAART) [1, 2]. It is projected that by 2015–2020, half of persons living with HIV infection in the United States will be 50 years of age or older [3, 4] and likely living with and managing other comorbid, chronic medical conditions that often accompany the aging process. These conditions include hypertension, diabetes, and for women, who make up half of all persons living with HIV infection worldwide, menopause [5]. For HIV-infected women taking effective ART, this longer survival translates into many women likely living beyond menopause well into their postmenopausal years. Yet large gaps exist in our understanding of the effect of HIV on the aging process for HIV-infected women as they approach and live through menopause. Prioritizing efforts to learn more about the potential impact of HIV on the lives of menopausal women are warranted in an effort to optimize care and treatment for older HIV-infected women.

**Figure 1:**
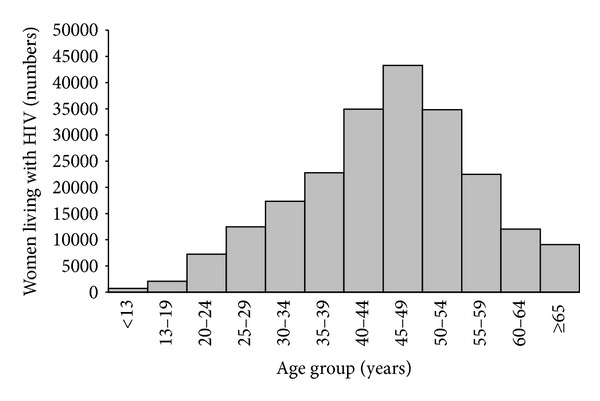
Estimated numbers of women living with HIV infection, 46 states, United States, 2011. Estimates were calculated based on data provided in [6].

**Figure 2:**
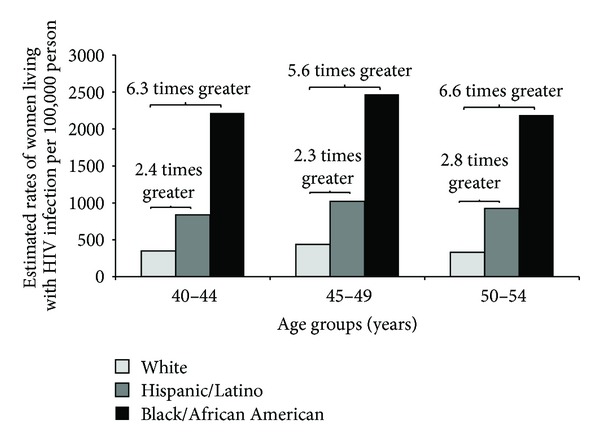
Estimated rates of women living with HIV infection, by age groups, 40–44, 45–49, and 50–54 years, among all women, by race/ethnicity in 46 states, United States, 2011. Estimates were calculated based on data provided in [6].

In 2011, an estimated 25% of U.S. adults and adolescents living with HIV infection were women; many were near or already into their menopausal years (Figure 1) [6]. Substantial racial/ethnic disparities have been noted among women living with HIV infection across all age groups; among women living with and newly diagnosed with HIV infection in 2011, black/African Americans and Hispanics/Latinas were disproportionately represented (Figure 2) [6–9]. Understanding the effects of HIV on women and menopause could contribute to efforts to understand and close HIV-related racial/ethnic disparities.

To date, there have been inconsistent reports regarding several areas related to HIV-infected women and menopause. HIV-infected women were reported to lose ovarian function earlier in life than HIV-uninfected women, leading to an earlier onset of menopause among HIV-infected women [10]. Over the short term, a menopausal transition is associated with an altered mood state and sexual dysfunction, both of which can affect quality of life for women [11, 12]. Over the long term, menopause accelerates the onset and progression of chronic diseases of aging, including cardiovascular disease, hypertension, diabetes, and reduced bone mineral density [13, 14]. All of these risks suggest a possible increased burden of disease for HIV-infected women if they enter menopause at an earlier age and are living longer lives on effective ART treatment [15]. Our objectives were to conduct a systematic review of the literature to summarize data regarding: (1) age of menopause among women living with HIV infection, and (2) immunological and virological response to ART in HIV-infected menopausal women. Our goal was to identify research gaps, including questions regarding racial/ethnic disparities, and inform future initiatives to ensure optimal medical management for older HIV-infected women.

## 2. Methods

We conducted a literature search using the Ovid Medline database from 1980 to August 2012 to identify relevant articles. The search was limited to articles published in English that described studies of menopausal transition among HIV-infected humans. We focused on two topics: (1) age at menopause and (2) immunological and virological response to ART across menopause. We felt that understanding the age at which HIV-infected women went through menopause would provide important, informative context about their responses to ART, especially related to possible menopause-related symptoms/side effects; this information may be helpful to clinical providers who care for HIV-infected, older women. Search strategies used the keywords “HIV” and “menopause.” Additional search terms—added to yield the highest number of possible articles—included (1) for age at menopause, “age,” “aging,” “age at menopause,” “age of menopause,” “early,” and “early menopause” and (2) for response to ART across menopause, “antiretroviral” (ARV), “HAART,” “ART,” “ARV,” “treatment,” “response,” “response to ART,” “CD4,” and “viral load.” Final original research articles included (1) original research articles that studied age at menopause in HIV-infected women, and (2) original research articles that examined the difference in response to ART across menopause in HIV-infected women.

We also reviewed research articles using the same search terms as above that compared ART responses among HIV-infected men and women aged ≤40 years with age ≥55 years to supplement our review of ART response across menopause among HIV-infected women. Some studies documented a wide median age range (39–53 years) for menopause among women living with HIV [16–18]. Most women have either entered or completed their menopausal transition by the age of 50 and almost all by the age of 55 [19]. HIV-infected women aged ≤40 years or aged ≥55 years may be characterized as premenopausal or postmenopausal, respectively, but documentation of menopausal status may vary depending on available clinical laboratory details reported in studies. Also, some studies have shown that immunological and virological response to ART do not differ by gender after adjusting for other factors, including age, race/ethnicity, and ART regimen [20–24].

## 3. Results

### 3.1. Final Articles Included in Review

**Figure 3:**
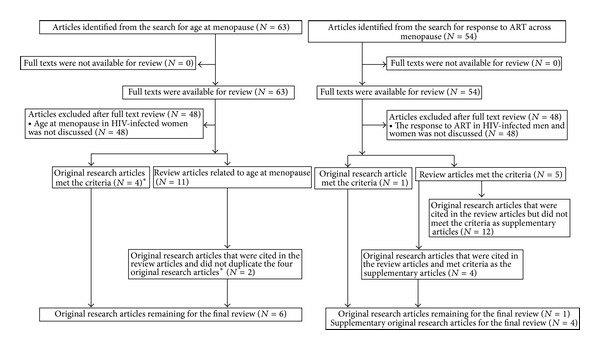
Flowchart of article selection and exclusion for the systematic review: HIV and menopause, 1980–2012.

The process for article selection and exclusion is summarized in Figure 1. For age at menopause, 63 articles were identified, of which four were original research articles [16, 18, 25, 26] and 11 were review articles [10, 15, 17, 27–34]. Forty-eight of 63 were excluded, because age at menopause in HIV-infected women was not discussed. In the 11 review articles, two more original research articles [17, 35] that did not duplicate the four original research articles [16, 18, 25, 26] were identified, for a total of six original research articles [16–18, 25, 26, 35] for the review of age at menopause in HIV-infected women (Figure 3).

For ART response among HIV-infected persons, 54 articles were identified. Of those, a single original research article [36] and five review articles [4, 28, 34, 37, 38] that reported response to ART in HIV-infected women were identified; 48 of 54 were excluded, because immunological response to ART in HIV-infected patients was not discussed. In the five review articles, 16 original research articles regarding response to ART in HIV-infected patients [24, 39–53] were also reviewed as possible supplementary articles. Of these, four original research articles [39–42] that contained useful information about the two age groups of HIV-infected men and women (ages ≤40 years and ≥55 years) were selected as supplementary articles for the final review. Overall, one article that enrolled HIV-infected women only and four supplementary original research articles (five total) [36, 39–42] were selected for the final review of response to ART among HIV-infected persons across menopause (Figure 1).

### 3.2. Age at Menopause

Age at menopause of HIV-infected women was reported in six studies [16–18, 25, 26, 35] (Table 1). Four studies were conducted in the United States with racially and ethnically diverse groups of participants (consistent with the United States' epidemiology of HIV among women) [16, 17, 26, 35], one in Brazil [25] and one in France [18]. In four of the six studies, the authors assessed median age at menopause in describing age at menopause [16–18, 25]; the remaining two studies assessed mean age at menopause [26, 35]. All six studies [16–18, 25, 26, 35] defined menopause as amenorrhea for 12 consecutive months [54].

Three of the six studies found no difference in age at menopause among HIV-infected [17, 18, 35] and HIV-uninfected women [36]. Cejtin et al. found no significant difference in the mean age at menopause between HIV-infected and HIV-uninfected women in the Women Interagency HIV Study (WIHS): 47.7 and 48.0 years, respectively [35]. The WIHS study participants were demographically similar to those in a study by Fantry et al. [17], in terms of race/ethnicity, level of education, socioeconomic status, and drug use, all of which have been reported to influence age at menopause [55]. The median age at menopause for HIV-infected women was 50 years (IQR: 49–53) in the Fantry et al. study [17] and 49 years (IQR: 40–50) in a study by de Pommerol et al. [18], similar to what has been reported with large multiethnic population-based samples of HIV-uninfected women (median age for menopause = 50–52 years) in the United States [56, 57] and Europe [58, 59].

In contrast, the other three studies [16, 25, 26] reported that menopause started at a younger age in HIV-infected women (Table 1). A single cohort study by Schoembaum et al. reported a significant lower median age at menopause in HIV-infected women (46.0 years, IQR: 39.0–49.0), compared with HIV-uninfected women (47.0 years, IQR: 44.5–48.0, *P* = 0.03) [16]. In an analysis using multivariate logistic regression with adjustment for age, these authors demonstrated that HIV-infected women were 73% more likely to experience premature menopause compared with HIV-uninfected women (OR: 1.73, 95% confidence interval [CI]: 1.08–2.80, *P* = 0.024) [16]. Two other studies from Ferreira et al. and Clark et al. that included only HIV-infected women reported median ages at menopause of 47.5 years (IQR not provided) [25] and 47 years (IQR: 32.0–57.0), respectively [26].

In one study included in this review, de Pommerol et al. examined premature menopause (onset before age 40 years) and reported that 12% of postmenopausal HIV-infected women sampled had experienced premature menopause [18]. This prevalence of premature menopause among HIV-infected women was higher than the reported prevalence of 1.1–6.3% among women in the general population of HIV-uninfected women enrolled in the American Study of Women's Health Across the Nation cohort, although the sampled populations were different demographically [60, 61]. Other reports have noted comparable mean ages at menopause between HIV-infected and HIV-uninfected women but found a disproportionate number of HIV-infected women with a younger median age for menopause [62, 63].

Three studies examined the association of CD4 cell count with age at menopause [18–20]. Schoembaum et al. reported that a CD4 cell counts of >500 cells/mm^3^ (OR: 0.19, 95% CI: 0.08–0.48, *P* = 0.001) and 200–500 cells/mm^3^ (OR: 0.35, 95% CI: 0.15–0.81, *P* = 0.015) was independently associated with a decreased risk of premature menopause compared with a CD4 cell count <200 cells/mm^3^ [16]. The median age of menopause was 42.5 years in HIV-infected women with CD4 cell counts of <200 cells/mm^3^, while the median age of menopause was 46.0 years in those with CD4 cell counts of 200–500 cells/mm^3^ and 46.5 years in those with CD4 cell counts of >500 cells/mm^3^ (*P* = 0.009) [16]. de Pommerol et al. reported that a CD4 cell count of <200 cells/mm^3^ at enrollment was also associated with an earlier onset of menopause, compared with a CD4 cell count of >350 cells/mm^3^ (HR: 2.25, 95% CI: 0.94–5.39, *P* = 0.069) [18]. Fantry et al. also showed no significant association between lower CD4 cell count and onset of menopause [17].

Although the same definition was used for menopause for all six studies, each used different methods for documenting age at menopause, and all evaluated menopause at a single time point [16–18, 25, 26, 35]. Evaluating the presence of menopause at only one time point could have led to an overestimation of its prevalence. Only the Cejtin et al. study [35] measured menopause using laboratory biologic markers, including follicle stimulating hormone (for diagnosing menopause, in addition to self-reported menopausal status, multiple consistently elevated serum FSH levels are useful as a laboratory marker [17] and can help distinguish menopause from other causes of amenorrhea [54, 64].) in addition to the women's self-report of amenorrhea (FSH). The other five studies [16–18, 25, 26] used only self-reported questionnaire data to document age at menopause and did not confirm menopause biologically. Thus, age at menopause reported in the five studies [16–18, 25, 26] could be different from the age when the women actually experienced menopause, as no laboratory verifications were obtained and misdiagnoses of menopause in HIV-infected women were possible.

### 3.3. Immunological and Virological Response to ART across Menopause

To evaluate response to ART in HIV-infected women and men, we reviewed studies that examined changes in CD4 cell counts (immunological response) and plasma HIV RNA viral loads (virological response) after initiation of ART [21, 36, 39, 40, 44, 49–51]. We identified only one study that examined response to ART in HIV-infected women with well-characterized menopause [36]. This study (Table 2) included 267 HIV-infected racially and ethnically diverse women (220 premenopausal and 47 postmenopausal) and demonstrated that the median change in absolute CD4 cell counts and percentages did not differ between pre-menopausal and post-menopausal women after two years of ART (260 versus 273 cells/mm^3^, *P* = 0.51; 11.0% versus 12.0%, *P* = 0.79) [36]. There were also no differences between pre-menopausal and post-menopausal women in the proportions achieving plasma HIV RNA viral loads <50 copies/mL after two years of HAART (75% versus 77%, *P* > 0.99; OR: 0.82, 95% CI: 0.36–1.89) [36]. These data suggest that both pre- and post-menopausal HIV-infected women compared with uninfected women respond equally well to ART during at least the first two years after initiation.

We also identified four supplementary original research articles regarding ART response among both men and women living with HIV infection (Table 3) [39–42]. Two studies [41, 42] included only ART-naïve HIV-infected men and women in the pre-menopausal and post-menopausal age ranges, whereas the two other studies [39, 40] included some ART-experienced patients. Three of the four supplementary articles analyzed data from European cohorts [39–41]. The fourth used data from the United States and Canada (North American AIDS Cohort Collaboration on Research and Design) and consisted of 83% male patients [42].

Regarding immunological response to ART, Manfredi and Chiodo found that both men and women aged ≥55 years who started ART had a significantly smaller increase in mean CD4 cell counts compared with persons aged ≤35 years (77 versus 114 cells/mm^3^, *P* = 0.0001) [39]. However, Knobel et al. and Althoff et al. concluded that there was no significant difference in immunological response between HIV-infected men or women comparing persons age ≤40 years with persons age ≥60 years [40, 42]. Finally, the Collaboration of Observational HIV Epidemiological Research in Europe (COHERE) study showed that HIV-infected men and women combined aged 55–59 years had a similar immunological response to ART compared with persons aged 30–39 years (HR: 0.97, 95% CI: 0.92–1.03, *P* = 0.31) [41]. However, HIV-infected women aged ≥60 years in the COHERE study were 7% less likely to experience immunological response compared with women aged 30–39 years (HR: 0.93, 95% CI: 0.87–0.98, *P* < 0.001) [41].

Regarding virological response to ART, both the Manfredi et al. and Knobel et al. studies reported that HIV-infected men and women aged ≤35 years [39] or aged ≤40 years [40] had no significant difference in either the decrease in mean plasma HIV RNA viral load or the number of patients achieving <50 copies/mL, compared with those aged ≥55 or aged ≥60, respectively (Table 3) [39, 40]; these studies were limited by small sample sizes. However, the COHERE study (>49,900 participants) showed that the probability of virological response was 24% higher in patients aged 55–59 years than those aged 30–39 years (HR: 1.24, 95% CI: 1.17–1.32, *P* < 0.001) [41]. In contrast, Althoff (>12,000 participants) showed that the hazard of achieving <500 copies/mL of HIV viral loads within two years after ART initiation in HIV-infected patients aged ≥60 years was 26% less than that in patients aged 18–29 years [42].

Overall, the outcomes of the four supplemental studies [39–42] were conflicting. Possible reasons include having used different age categories for comparison, variation in study sample size, and lack of data for women only. Moreover, none of the four studies, which sampled both men and women with HIV infection, stratified data by sex, and menopause status to permit comparison with the Patterson et al. study [36], which consisted solely of women. The number of men was significantly greater than that of women for studies in Table 3 and could have affected study outcomes. Some studies, not included in our review because data on menopause were not reported, suggest that immunological and/or virological responses to ART differ by gender and among all age groups after adjusting for other factors [22, 65, 66].

## 4. Discussion

Our review revealed that current data on age at menopause and the effect of menopause on the response to ART for HIV-infected women are conflicting. Part of the challenge is that existing studies have not adequately distinguished the unique contribution, if any, of HIV infection or its consequences such as lower CD4 cell count [16, 18], from the contribution of other risk factors for early menopause irregular menses [15], such as drug use [18], tobacco smoking [67], black/African American race [18], fewer years of education [16, 57], and lower body mass index (BMI) [54, 59], all of which are common among persons living with HIV infection. Many current reports have been limited to cohorts of HIV-infected persons only [18, 56, 59, 68–70]. In addition, some conflicting data (in studies with small sample sizes) suggest that in contrast to women in the general population, age at menopause among HIV-infected women may not be associated with current smoking [16], ethnicity [16], or fewer years of education [18, 54]. Additionally, although women with more advanced HIV infection and potentially lower BMI or wasting may be more likely to have amenorrhea, the association between BMI and the onset of menopause is inconsistent [56, 71] or absent [59] in the limited number of studies of HIV-infected women that have examined this association.

Also, more consistency regarding the definition of menopause, including FSH biological measurements, is warranted for future studies, because not all amenorrhea is menopause. Menopause is usually defined as the cessation of menses (amenorrhea) in women for ≥12 consecutive months with symptoms suggestive of menopause and in which other causes of amenorrhea have been ruled out and/or the FSH level is elevated. Amenorrhea, which is defined as missing menstrual periods for at least three consecutive months [63], has been reported commonly among HIV-infected women and has been attributed to wasting [72] or anovulation [73]. Cejtin et al. reported that HIV infection was significantly associated with prolonged amenorrhea from causes other than menopause (OR: 3.16, 95% CI: 1.26–7.95, *P* = 0.02) when adjusted for age [54]. Cejtin et al. reported that only 74.6% of HIV-infected women who were age 45 years or older and had prolonged amenorrhea were truly menopausal [54], while nearly 90% of HIV-uninfected women agedd ≥45 years with prolonged amenorrhea were truly menopausal [74]. Presence of menses may also be affected by use of ART; ART was significantly associated with having higher serum FSH among HIV-infected women compared with uninfected women (*P* < 0.05) [16]. Use of ART (HR: 0.48, 95% CI: 0.32–0.71, *P* = 0.003) and higher CD4 cell counts (*P* = 0.007) have been linked to a lower incidence of amenorrhea [75]. HIV-infected women who have HIV-related hypothalamic amenorrhea, especially those taking ART, may experience a return of menses once CD4 cell counts increase [75].

Menopause results in decreased ovarian synthesis of estrogen; estrogen and progesterone can effect HIV replication in peripheral blood mononuclear cells [76]. In animals, estrogen deficiency reduces the percentage of T cells in bone marrow [77]. Among HIV-uninfected women, post-menopausal women have lower CD4 cell counts than pre-menopausal women [78]. Estrogen deficiency associated with menopause and normal reduction in thymic tissue that accompanies aging [79] could affect CD4 cell recovery and HIV replication. Thus, post-menopausal women may respond differently to ART compared with pre-menopausal women.

Regarding the effect of menopause on ART response, we were unable to compare the Patterson et al. study [36] to any other report, because no other studies were conducted with only HIV-infected women. Although it is difficult to disentangle the effect of aging on response to ART, we assessed data from the supplementary articles (both women and men) in order to compensate for the paucity of specific data (women only) of the effect of menopause on response to ART. Data from the supplementary articles were inconsistent. Some data suggest that younger persons experience better immunological and/or virological responses to ART [44, 50, 52]. The COHERE study showed that immunological response to ART was similar in the patients aged 55–59 years but poorer in those aged ≥60 years, compared with the patients who were younger than the average age at menopause [41]. This finding may imply that the effects of aging on CD4 cell count recovery are multifactorial, resulting from both normal age-related immunosenescence and by the negative effects of estrogen deficiency that occurs with menopause [80, 81]. If a low CD4 cell count increases risk of early menopause, then the effects of HIV infection and menopause would be compounded; earlier diagnosis of HIV and initiating ART treatment before CD4 cells are <200 cells/mm^3^ would be warranted for women approaching menopause.

## 5. Conclusion/Recommendations for Next Steps

Understanding the effects of HIV infection on age at menopause and on response to ART is important for ensuring the best possible care for the expanding cohort of women living with HIV who approach and live through menopause and are taking ART. This information may also help inform clinicians and researchers to assess whether ART for middle-aged women with HIV infection should be modified across menopause. Advanced HIV infection is a known independent risk factor for early mortality as well as both cardiovascular disease and decreased bone mineral density. Earlier onset of menopause is also associated with increased mortality [82, 83] and both decreased bone mineral density [84] and cardiovascular disease [85].

Our review highlights research deficits in our understanding of the effects of HIV infection on age at menopause and opportunities for future work. Ideally, studies of menopause in HIV-infected women should include sufficient numbers of women, both HIV-infected and HIV-uninfected, who are representative of the racially and ethnically diverse women living with or at risk of HIV infection in the United States. Such studies should follow women across menopause and capture data on the growing number of factors recognized to be related to age of menopause, including race/ethnicity, tobacco smoking, drug use, level of education, BMI, use of ART, CD4 cell counts, and HIV viral loads. Furthermore, a combination of questionnaires and laboratory measurement of serum FSH levels will improve classification of menopause in women, especially HIV-infected women for whom amenorrhea is highly prevalent. Additional data may also be helpful for clinicians managing HIV-infected women with chronic medical conditions that also disproportionately affect older black/African American and Hispanic/Latina women of color, such as diabetes and hypertension [86, 87].

Today, over 30 years since HIV was first identified, the number of women living with HIV infection, taking ART, and living longer and healthier lives is expanding. Epidemiological studies of older, menopausal women living with HIV infection are needed to address questions raised as this epidemiologic landscape changes. Such studies will provide important data that will help to optimize care for the growing numbers of post-menopausal women living with HIV infection.

## Figures and Tables

**Table 1:** Studies available through 2012 evaluating age at menopause in HIV-infected women.

Authors (year)	Country	Number of women for analysis	Percent of participants who were black/African American or Hispanic/Latina	HIV status	*N*	Number of women with menopause	Age at onset of menopause (years)	*P* value
Clark et al. (2000) [26]	United States	52	43% (black/AA) 15% (Hispanic)	Infected Uninfected	52 0	26 (50.0%) NA	47 (IQR 32–57) (mean) NA	NA
Cejtin et al. (2004) [35]	United States	1335	NR	Infected Uninfected	1063 272	NR NR	47.7 (mean) 48.0	NS
Fantry et al. (2005) [17]	United States	120	95% (black/AA)	Infected Uninfected	120 0	NR NA	50.0 (IQR 49.3–53.0) (median) NA	NA
Schoenbaum et al. (2005) [16]	United States	571	49% (black/AA) 40% (Hispanic)	Infected Uninfected	302 269	62 (20.5%) 40 (14.9%)	46.0 (IQR 39.0–49.0) 47.0 (IQR 44.5–48.0)	0.03
Ferreira et al. (2007) [25]	Brazil	251	NA	Infected Uninfected	96 155	NR NR	47.5 (median) NR	NR
de Pommerol et al. (2011) [18]	France	404	NA	Infected Uninfected	404 0	69 (17.1%) NA	49 (IQR 40–50) (median) NA	NA

AA: African American, IQR: interquartile range, NA: not applicable, NR: not reported, NS: not significant.

**Table 2:** Studies available through 2012 evaluating response to ART across menopause in HIV-infected women.

Authors (year)	Study design (followup)	Menopause status	Number of women (total sample; *n* = 267)	Percent of total participants who were black/African American or Hispanic/Latina	CD4 cell counts in women after ART			HIV viral loads in women after ART		
					Measure	Results	*P* value	Measure	Results	*P* value
Patterson et al. (2009) [36]	Cohort (2 years)	Pre Post	220 47	61% (black/AA) 17% (Hispanic)	Increase in median CD4 from baseline	260 (11.0%) 273 (12.0%)	0.51	Women achieving <50 copies/mL	75% 77%	>0.99
								Odds ratio of achieving <50 copies/mL for premenopausal women	0.82 (0.36–1.89)	NS

AA: African American, NS: not significant, Pre: premenopausal, Post: postmenopausal.

**Table 3:** Supplemental list of studies evaluating response to ART in HIV-infected persons (men and women), through 2012.

Authors (year)	Study design (followup)	Age groups (years)	Number of patients	Number of women	CD4 cell counts in persons after initiating ART			HIV viral loads in persons after ART		
					Measure	Results	*P* value	Measure	Results	*P* value
Manfredi and Chiodo (2000) [39]	Case-control (1 year)	≤35 ≥55	84 21	29 (34.5%) 8 (38.1%)	Patients with CD4 increase ≤20 cells/mm^3^ or ≤10% from baseline (12-month followup)	4 (4.8%) 5 (23.8%)	0.02	Decrease in mean viral load (copies/mL)	31,225 (98.7%) 49,325 (98.4%)	NS
					Increase in mean CD4 from baseline (% of increase) (12-month followup)	114 (49.4%) 77 (36.3%)	0.0001	Patients achieving <50	62 (73.8%) 15 (71.4%)	NS
Knobel et al. (2001) [40]	Cohort (2 years)	≤40 ≥60	671 28	219 (32.6%) 9 (32.1%)	Increase in mean CD4 from baseline (24-month followup)	196 (SD 100) 228 (SD 145)	NS	Patients achieving <50	342 (50.9%) 19 (66.7%)	NS
COHERE Study, Sabin (2008) [41]	Cohort (5 years)	30–39 55–59 ≥60 All ages	22,410 1,656 1,613 49,921	6239 (27.8%) 303 (18.3%) 320 (19.8%)	HR of immunological response (defined as CD4 increase of >100 cells/*μ*L)	REF 0.97 (0.92–1.03) 0.93 (0.87–0.98)	0.31 <0.001	HR of virological response (defined as HIV RNA <50 copies/mL)	REF 1.24 (1.17–1.32) 1.18 (1.12–1.26)	<0.001 <0.001
Althoff et al. (2010) [42]	Cohort (2 years)	18–29 30–39 ≥60 All ages	1,342 3,930 598 12,196	320 (24.8%) 767 (19.5%) 50 (8.4%)	HR of CD4 increase of at least 100 cells/mm^3^ within 2 years of ART initiation	REF 0.71 (0.89–1.05) 1.05 (0.92–1.20)	NR	HR of achieving <500 copies/mL within 2 years of ART initiation	REF 0.92 (0.85–1.00) 0.74 (0.65–0.85)	NR

ART: antiretroviral treatment, HR: hazard ratio, NR: not reported, NS: not significant, REF: referent, yrs: years.
